# Severe Antenatal Presentation of a Novel Dnase2 Mutation in a Preterm Omani Neonate: Expanding the Clinical Spectrum of an Ultra-Rare Interferonopathy

**DOI:** 10.7759/cureus.111043

**Published:** 2026-06-17

**Authors:** Ruqaiya Al Jashmi, Marya Al Barumi, Safiya Al- Abrawi, Alyaa Al Mughairy, Aiman Al Jabri

**Affiliations:** 1 Pediatric Rheumatology, Royal Hospital, Muscat, OMN; 2 Pediatrics, Oman Medical Specialty Board, Muscat, OMN; 3 Child Oncology, Oman National Oncology Center, Muscat, OMN; 4 Child Health, Sultan Qaboos University Hospital, Muscat, OMN

**Keywords:** aids, autoinflammatory disease, dnase 2 deficiency, type i interferonopathies, vasculitis

## Abstract

Dnase2 deficiency is an ultra-rare type I interferonopathy resulting from impaired intracellular DNA degradation, leading to neonatal cytopenias and systemic inflammation. Whole-exome sequencing enables definitive diagnosis, and Janus kinase inhibitors have emerged as targeted immunomodulatory therapy.

This report describes a preterm infant with an early and severe presentation of DNASE2 deficiency, manifesting antenatally with fetal hydrops, intrauterine growth restriction, and cardiomyopathy. Postnatally, the infant developed persistent pancytopenia, hepatosplenomegaly, cholestatic jaundice, and hemophagocytic lymphohistiocytosis-like features, including marked hyperferritinemia. Whole-exome sequencing identified two novel heterozygous pathogenic variants in the *DNASE2* gene. Initiation of Janus kinase inhibitor therapy (ruxolitinib) was challenging due to the patient’s young age and low body weight; however, targeted treatment was associated with significant clinical improvement, including improved growth parameters and normalization of inflammatory and hemophagocytic lymphohistiocytosis (HLH)-associated markers during outpatient follow-up.

This review underscores the severe antenatal and early postnatal manifestations of a novel Dnase2 deficiency, including fetal anemia, hydrops fetalis, and HLH-like inflammation in a preterm neonate. The observed clinical improvement following Janus kinase inhibitor therapy suggests that early targeted intervention may improve outcomes in this ultra-rare interferonopathy.

## Introduction

Type I interferonopathies are a heterogeneous group of monogenic autoinflammatory disorders characterized by inappropriate activation of type I interferon (IFN-I) signaling pathways [1-5]. These conditions are characterized by a persistent upregulation of interferon-stimulated genes (ISGs), commonly referred to as the interferon signature, resulting in chronic innate immune activation and systemic inflammation [1-6].

Defects in nucleic acid degradation represent a major pathogenic mechanism underlying type I interferonopathies. Impaired clearance of endogenous DNA or RNA results in persistent activation of innate immune sensors and chronic production of type I interferons [2,5-8].

Dnase2 deficiency is an exceptionally rare autosomal recessive type I interferonopathy caused by biallelic pathogenic variants in the Dnase2 gene located on chromosome 19p13.13 [8-10]. The Dnase2 gene encodes deoxyribonuclease II (DNase II), a lysosomal endonuclease responsible for degradation of DNA derived from apoptotic cells and nuclei extruded during erythropoiesis [9,10]. Defective DNase II activity leads to an accumulation of undegraded DNA within lysosomes and activation of intracellular DNA-sensing pathways, particularly the cyclic GMP-AMP synthase-stimulator of interferon genes (cGAS-STING) axis, resulting in a sustained production of type I interferons and pro-inflammatory cytokines [7,11,12].

The reported patients presented with a broad spectrum of manifestations, including severe neonatal anemia, thrombocytopenia, hepatosplenomegaly, cholestatic hepatitis, recurrent inflammatory episodes, membranoproliferative glomerulonephritis, liver fibrosis, vasculitic skin lesions, and progressive deforming arthropathy [11]. Subsequent reports expanded the phenotypic spectrum to include recurrent fevers, autoimmune manifestations, growth failure, osteopenia, joint contractures, oral ulcers, positive autoantibodies, hemophagocytic lymphohistiocytosis, inflammatory bowel disease, and a marked interferon signature [13,14].

Advances in the understanding of interferon-mediated disease mechanisms have led to the use of Janus kinase (JAK) inhibitors as targeted therapy in selected type I interferonopathies [14,15]. Limited published data suggest that JAK inhibition may be effective in Dnase2 deficiency by suppressing downstream interferon signaling. In particular, treatment with baricitinib has been associated with clinical and immunological improvement in a patient with genetically confirmed Dnase2 deficiency [14]. However, clinical experience remains extremely limited, particularly in neonates and young infants.

Here, we report the first genetically confirmed case of Dnase2 deficiency in the Middle East and describe a severe antenatal-onset presentation in a preterm infant. This case expands the clinical spectrum of Dnase2 deficiency and provides further evidence supporting the role of early genetic diagnosis and targeted JAK inhibitor therapy in this ultra-rare condition.

## Case presentation

**Figure 1:**
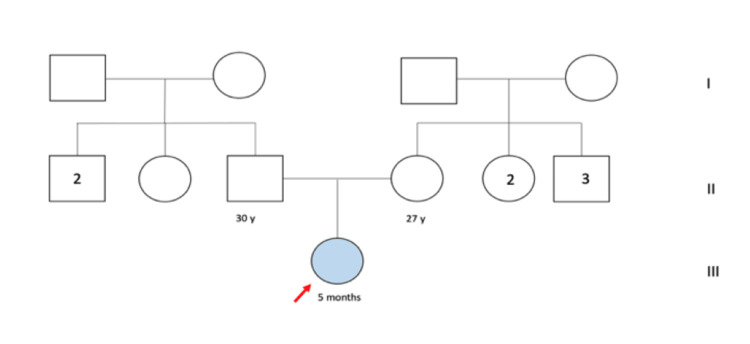
Family Pedigree I: First Generation; II: Second Generation; III: Third Generation. Arrow: affected child with Dnase 2 deficiency.

**Figure 2:**
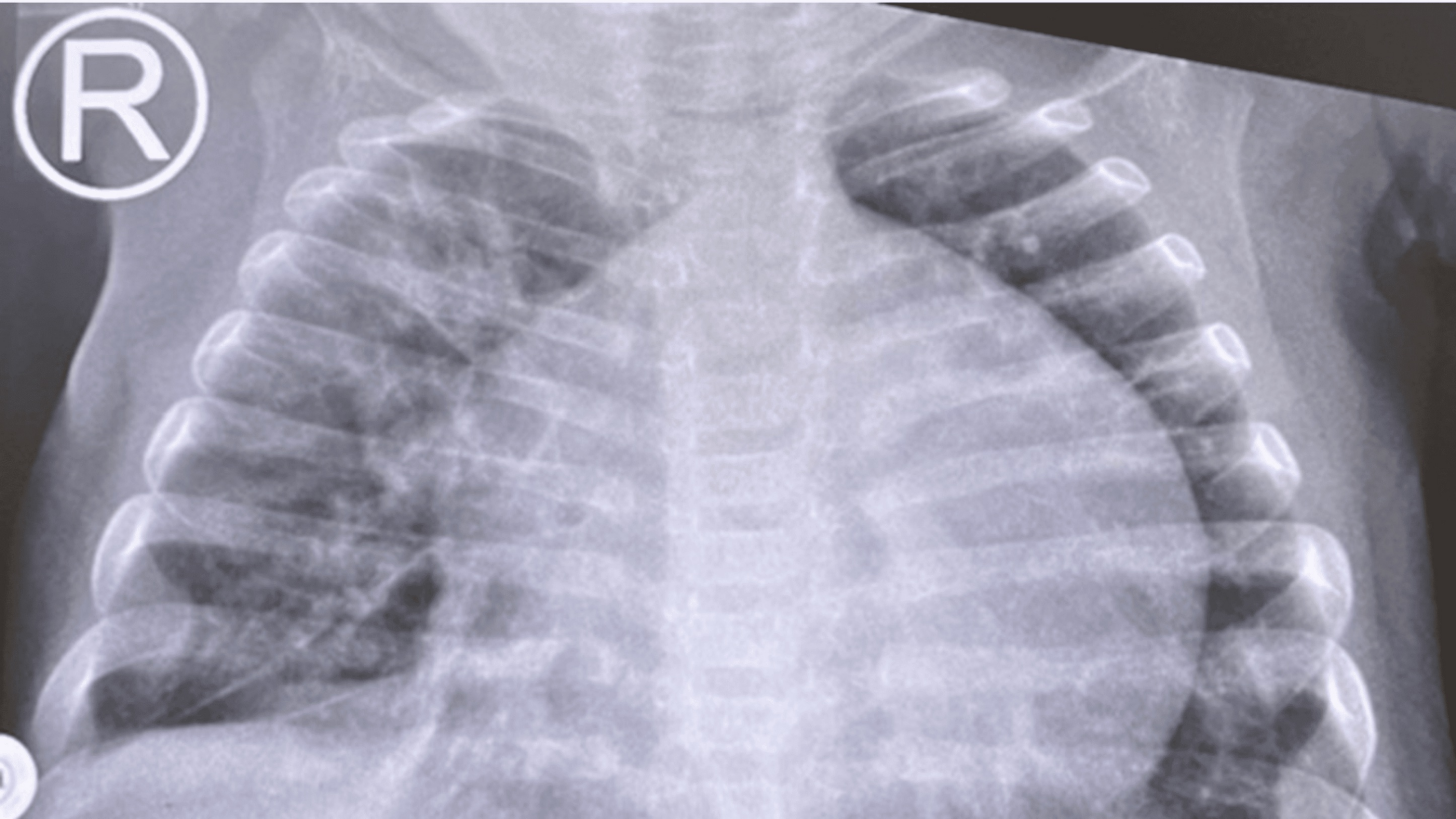
Chest X-ray at the day of admission shows cardiomegaly and right-side infiltrate

A preterm female infant was delivered at a gestation of 30 weeks to non-consanguineous healthy parents following an attempted intrauterine transfusion for severe fetal anemia (Figure 1). Antenatal ultrasonography demonstrated symmetric intrauterine growth restriction and hypertrophic cardiomegaly. At birth, Apgar scores were reassuring; however, the neonate soon developed recurrent apnea and bradycardia, necessitating escalation of respiratory support from non-invasive ventilation to endotracheal intubation and mechanical ventilation. His chest X-ray showed marked cardiomegaly and right-side infiltrations (Figure 2).

Birth anthropometric measurements revealed a weight of 950 g (<3rd percentile), length of 35 cm (<3rd percentile), and head circumference of 27 cm (25th-50th percentile). On physical examination, the infant was noted to have marked jaundice and hepatosplenomegaly. A single umbilical artery was present, and no dysmorphic features were identified.

Initial laboratory investigations demonstrated respiratory acidosis and transient hypoglycemia, in addition to severe pancytopenia, with haemoglobin of 8.2 g/dL, platelet count of 12×10⁹/L, white blood cell count of 1.3×10⁹/L, and an absolute neutrophil count of 0. Liver biochemistry revealed significant transaminitis and conjugated hyperbilirubinemia (total bilirubin 300 µmol/L, conjugated fraction 255 µmol/L), consistent with cholestatic liver disease. The coagulation profile was abnormal, and ferritin levels were markedly elevated (6886 ug/L). An extensive infectious evaluation, including TORCH screening (Toxoplasmosis, Rubella, Cytomegalovirus (CMV) and Herpes), yielded negative results (Table 1).

**Table 1:** Laboratory results of the patient with Dnase 2 Deficiency before starting Ruxolitinib

Test Name	Value	Unit	Reference range
Haemoglobin	8.2	g/dL	12.5-19.5
Platelet count	12	10^9^/L	150-450
White Blood Cells	1.3	10^9^/L	6-22
Neutrophils	0.0	10^9^/L	4.5-12
Ferritin	6886	µg/L	10-291
Triglycerides	2.37	mmol/L	-
Fibrinogen	1.25	g/L	2.25-3.41
Bilirubin Total	300	umol/L	0-100
Conjugated Bilirubin	255	umol/L	0-5
Alanine transaminase (ALT)	210	IU/L	10-40
Aspartate aminotransferase (AST)	615	IU/L	10-34

**Figure 3:**
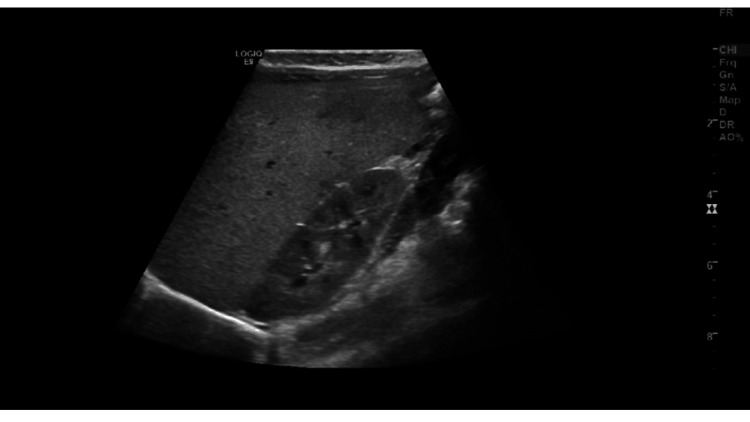
Abdomen ultrasound (AUS) shows enlarged liver with increased parenchymal echo texture. No focal lesion.

**Figure 4:**
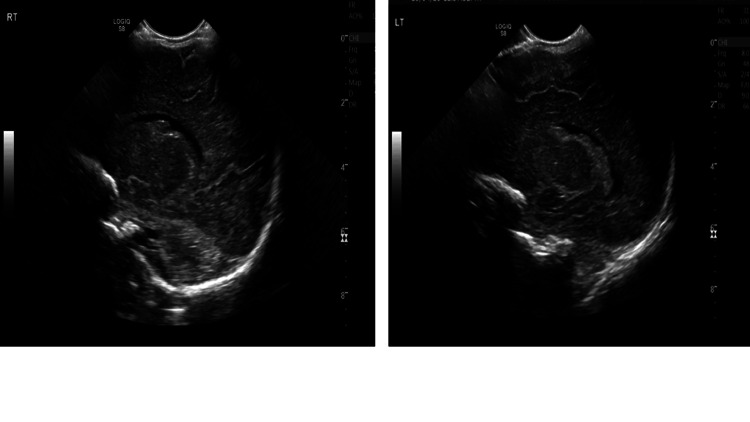
Head ultrasonography of the baby Rt: Right-side intraventricular hemorrhage (IVH). Lt: Left-side intraventricular hemorrhage (IVH).

Abdominal ultrasonography demonstrated hepatosplenomegaly with coarse hepatic echotexture (Figure 3). Cranial ultrasonography identified a grade I bilateral intraventricular hemorrhage (Figure 4). Transthoracic echocardiography revealed a large patent ductus arteriosus, which was managed conservatively with medical therapy.

During the neonatal intensive care unit (NICU) course, the patient required multiple transfusions of blood products due to persistent cytopenias. The combination of sustained pancytopenia, hepatosplenomegaly, hyperferritinemia, hypofibrinogenemia, and liver dysfunction raised concern for hemophagocytic lymphohistiocytosis (HLH). However, the patient did not fulfill the HLH 2004 criteria. The infant was treated with intravenous immunoglobulin for ongoing microangiopathic hemolytic anemia. Supportive therapy with ursodeoxycholic acid and vitamin K was initiated to address cholestasis and coagulopathy.

Whole-exome sequencing identified two novel heterozygous variants in the Dnase2 gene (p.Gly48AlafsTer49 and p.Met1), consistent with a compound heterozygous state and confirming a diagnosis of Dnase2 deficiency, an autosomal recessive autoinflammatory disorder characterized by immune dysregulation and cytopenias (Table 2).

**Table 2:** Whole-exome sequencing showing heterozygous mutation of Dnase 2 gene

Gene	Variant Coordinates	Amino Acid Change	SNP Identifier	Zygosity	In Silico Parameters	Allele Frequencies	Type and Classification
DNASE2	NM_001375.2:c.141_142del	p.(Gly48AlafsTer49)	N/A	Heterozygous	PolyPhen: N/AAlign-GVGD: N/ASIFT: N/AMutationTaster: N/AConservation_nt: —Conservation_aa: —	gnomAD: -ESP: -1000G: -CentoMD: -	Frameshift Uncertain significance (Class 3)
DNASE2	NM_001375.2:c.2T>C	p.(Met1?)	N/A	Heterozygous	PolyPhen: N/AAlign-GVGD: N/ASIFT: N/AMutationTaster: N/AConservation_nt: weakConservation_aa: —	gnomAD: 0.0000064ESP: -1000G: =CentoMD: -	Start-lost Uncertain significance (Class 3)

In light of ongoing pancytopenia and hepatic dysfunction, treatment with a Janus kinase inhibitor (Ruxolitinib) was considered during the NICU admission but deferred because of extreme prematurity, very low body weight, and limited published safety data in this age group. At three months of age, the infant continued to exhibit persistent pancytopenia and cholestatic liver disease, prompting initiation of Ruxolitinib therapy. Subsequent follow-up demonstrated progressive improvement in hematologic parameters and resolution of cholestatic liver dysfunction (Table 3).

**Table 3:** Laboratory results of the patient with Dnase 2 Deficiency after starting Ruxolitinib

Test Name	Value	Unit	Ref-Range
Haemoglobin	12.2	g/dL	12.5-19.5
Platelet count	361	10^9^/L	150-450
White Blood Cells	4.0	10^9^/L	6-22
Neutrophils #	0.9	10^9^/L	4.5-12
Ferritin	266	µg/L	10-291
Bilirubin Total	4	umol/L	0-100
Conjugated Bilirubin	0	umol/L	0-5
Alanine Transaminase (ALT)	68	IU/L	10-40
Aspartate Aminotransferase (AST)	69	IU/L	10-34

## Discussion

This report describes the first genetically confirmed case of Dnase2 deficiency from the Middle East and represents, to our knowledge, the earliest and most severe phenotypic presentation reported to date. Unlike previously described patients, our case manifested antenatally with severe fetal anemia, symmetric intrauterine growth restriction, and hypertrophic cardiomyopathy, underscoring the critical role of DNase II in fetal hematopoiesis and immune regulation. Postnatally, the clinical features closely resembled those described by Rodero and Crow [8], including persistent pancytopenia, hepatosplenomegaly, and cholestatic hepatitis. However, in contrast to earlier reports in which neonatal anemia improved within the first weeks of life before recurring later in childhood, our patient demonstrated sustained pancytopenia and ongoing cholestatic liver disease at three months of age. This suggests a more severe disease course and a limited capacity for early hematologic recovery.

Previously reported patients developed additional organ manifestations later in childhood, including recurrent febrile episodes, immune-complex-mediated glomerulonephritis, insulin-dependent diabetes mellitus, and treatment-refractory non-destructive arthropathy [8]. While our patient has not yet developed these features due to early diagnosis and young age, long-term surveillance remains essential given the progressive nature of Dnase2-related disease.

Dnase2 deficiency has also been associated with early-onset autoinflammatory disease resembling HLH, as described by Hong et al. [14]. Our patient exhibited several HLH-like features, including hyperferritinemia, hypofibrinogenemia, cytopenias, and liver dysfunction, reinforcing the importance of considering monogenic interferonopathies in neonates with HLH-like presentations and negative infectious evaluations.

Genetic analysis in our case revealed two novel heterozygous pathogenic variants in Dnase2: a frameshift mutation (p.Gly48AlafsTer49) and a start-loss variant (p.Met1?), confirming compound heterozygosity. Both variants are predicted to result in complete loss of DNase II function through a premature truncation or failure of translation initiation. In contrast, previously reported Dnase2 mutations were predominantly homozygous missense variants or in-frame deletions that likely retain partial enzymatic activity. This distinction may explain the exceptionally early onset and severity observed in our patient.

Importantly, this case highlights that Dnase2 deficiency can occur in children born to non-consanguineous parents and should be considered even in sporadic presentations. Therapeutic experience in Dnase2 deficiency remains extremely limited. The most comprehensive report to date by Hong et al. demonstrated that treatment with the JAK1/2 inhibitor baricitinib resulted in the normalization of inflammatory markers, recovery of blood counts, and clinical improvement, supporting the central role of type I interferon signaling in disease pathogenesis [14]. Other reported cases primarily received supportive or conventional immunosuppressive therapy, with limited efficacy [8]. Initiation of JAK inhibition in our patient posed significant challenges due to extreme prematurity, very low birth weight, and the absence of published safety data in this age group. Nevertheless, Ruxolitinib was initiated at three months of age with careful dose escalation and close monitoring. Treatment resulted in sustained improvement of pancytopenia, normalization of liver enzymes, and overall clinical stabilization, without significant adverse effects. To our knowledge, this represents the youngest patient with Dnase2 deficiency treated successfully with a JAK inhibitor.

## Conclusions

Dnase2 deficiency should be considered in neonates and infants presenting with antenatal or early postnatal manifestations such as fetal anemia, intrauterine growth restriction, pancytopenia, hepatosplenomegaly, and cholestatic jaundice, particularly when infectious and acquired causes have been excluded. The disease may closely mimic hemophagocytic lymphohistiocytosis, emphasizing the importance of early genetic testing to establish a definitive diagnosis and guide targeted therapy. This case highlights the complexities of initiating JAK inhibitor therapy in extremely young, preterm infants with limited safety data. Despite these challenges, treatment with ruxolitinib in our patient resulted in marked clinical and laboratory improvement, supporting its role as a potentially disease-modifying therapy in Dnase2 deficiency. Early recognition and timely intervention with targeted immunomodulatory therapy may significantly improve outcomes and reduce exposure to prolonged empiric treatments.
